# Brief cognitive behavioral therapy in primary care: a hybrid type 2 patient-randomized effectiveness-implementation design

**DOI:** 10.1186/1748-5908-7-64

**Published:** 2012-07-11

**Authors:** Jeffrey A Cully, Maria E A Armento, Juliette Mott, Michael R Nadorff, Aanand D Naik, Melinda A Stanley, Kristen H Sorocco, Mark E Kunik, Nancy J Petersen, Michael R Kauth

**Affiliations:** 1 Houston VA HSR&D Center of Excellence, Michael E. DeBakey VA Medical Center, Houston, TX, USA; 2 VA South Central Mental Illness Research, Education and Clinical Center (a virtual center), USA; 3 Department of Psychiatry and Behavioral Sciences, Baylor College of Medicine, Houston, TX, USA; 4 Department of Medicine/Health Services Research, Baylor College of Medicine, Houston, TX, USA; 5 Oklahoma Veterans Affairs Medical Center, Oklahoma City, OK, USA; 6 Department of Geriatrics, University of Oklahoma Health Sciences Center, Oklahoma City, OK, USA

**Keywords:** Primary care, Hybrid effectiveness-implementation designs, Cognitive behavioral therapy, Mental health, Veterans, Anxiety, Depression

## Abstract

**Background:**

Despite the availability of evidence-based psychotherapies for depression and anxiety, they are underused in non-mental health specialty settings such as primary care. Hybrid effectiveness-implementation designs have the potential to evaluate clinical and implementation outcomes of evidence-based psychotherapies to improve their translation into routine clinical care practices.

**Methods:**

This protocol article discusses the study methodology and implementation strategies employed in an ongoing, hybrid, type 2 randomized controlled trial with two primary aims: (1) to determine whether a brief, manualized cognitive behavioral therapy administered by Veterans Affairs Primary Care Mental Health Integration program clinicians is effective in treating depression and anxiety in a sample of medically ill (chronic cardiopulmonary diseases) primary care patients and (2) to examine the acceptability, feasibility, and preliminary outcomes of a focused implementation strategy on improving adoption and fidelity of brief cognitive behavioral therapy at two Primary Care-Mental Health Integration clinics. The study uses a hybrid type 2 effectiveness/implementation design to simultaneously test clinical effectiveness and to collect pilot data on a multifaceted implementation strategy that includes an online training program, audit and feedback of session content, and internal and external facilitation. Additionally, the study engages the participation of an advisory council consisting of stakeholders from Primary Care-Mental Health Integration, as well as regional and national mental health leaders within the Veterans Administration. It targets recruitment of 320 participants randomized to brief cognitive behavioral therapy (n = 200) or usual care (n = 120). Both effectiveness and implementation outcomes are being assessed using mixed methods, including quantitative evaluation (*e.g.*, intent-to-treat analyses across multiple time points) and qualitative methods (*e.g.*, focus interviews and surveys from patients and providers). Patient-effectiveness outcomes include measures of depression, anxiety, and physical health functioning using blinded independent evaluators. Implementation outcomes include patient engagement and adherence and clinician brief cognitive behavioral therapy adoption and fidelity.

**Conclusions:**

Hybrid designs are needed to advance clinical effectiveness and implementation knowledge to improve healthcare practices. The current article describes the rationale and challenges associated with the use of a hybrid design for the study of brief cognitive behavioral therapy in primary care. Although trade-offs exist between scientific control and external validity, hybrid designs are part of an emerging approach that has the potential to rapidly advance both science and practice.

**Trial registration:**

NCT01149772 at http://www.clinicaltrials.gov/ct2/show/NCT01149772

## Background

Traditional cognitive behavioral therapy (CBT), consisting of 12 to 16 sessions over three to six months of treatment, is an efficacious treatment for depression and anxiety [1-5]. Much of the research on CBT has been conducted in academic trials and within specialty mental healthcare settings [6,7]. Less is known about the utilization of brief CBT (bCBT), consisting of four to six sessions over a maximum of four months, and its ability to be adopted within non-mental healthcare settings such as primary care. Treatment modalities like bCBT can dramatically enhance the reach of mental illness therapy, given the prevalence and morbidity of depression and anxiety among medically ill patients.

Although psychotherapies such as CBT improve patient outcomes, such treatments are infrequently used within integrated healthcare settings, especially in non-specialty mental healthcare settings such as primary care [8-11]. In an effort to improve the availability of psychotherapy, the Department of Veterans Affairs (VA) issued the Uniform Mental Health Services Handbook in 2008 [12], which mandated that VA medical centers embed mental health services into primary care settings and make available evidence-based psychotherapies for all veterans with depression and anxiety. Since 2008, the VA has promoted a national dissemination program called Primary Care-Mental Health Integration (PC-MHI) for all its hospitals and large community-based clinics and has also dedicated significant resources toward clinician training in traditional evidence-based psychotherapies, including CBT, using a 12- to 16-session approach [4,13,14].

Unfortunately, mental health providers in the primary care setting face challenges to implementation of traditional evidence-based psychotherapies. Evidence suggests that patients treated in primary care are distinct from specialty mental healthcare patients (*e.g.*, present with physical health concerns and fewer chronic and severe mental health difficulties) and require modifications to traditional approaches [15,16]. Data also suggest that primary care patients may benefit from mental health interventions that are less intensive and focus treatment around physical as well as emotional health concerns [17-19]. These patients may also prefer and engage more fully in care co-located in primary care [20,21]. Lastly, VA PC-MHI providers require assistance to determine how best to modify traditional evidence-based therapies to better align with the practice demands of the primary care setting, which are increasingly involving collaborations between mental and physical health providers (*e.g.*, medical home or the Patient-Aligned Care Team initiative within the VA). For example, in the VA PC-MHI program, clinicians are asked to provide evidence-based care (*e.g.*, 12–16 sessions of CBT) within an integrated primary care model that encourages psychotherapy services in the range of four to six sessions of care [11,22,23]. Presently, mental health providers in primary care are struggling to adapt traditional evidence-based approaches, and they need data to support their delivery of high-quality care.

Initial studies provide evidence that bCBT is an effective treatment within primary care for depression, panic, and generalized anxiety disorders [17,18,24-27]. Although studies support the general efficacy of bCBT, additional effectiveness data are needed to determine the impact of these treatments under real-world conditions and to understand the potential for bCBT to be integrated within primary care.

### Using blended effectiveness-implementation designs to improve the provision of evidence-based psychotherapy

It is imperative that research focus on increasing use of evidence-based interventions in frontline practice [28]. Unfortunately, significant time and resources are required by the traditional sequential scientific approach that moves from clinical efficacy to effectiveness and then implementation [29], and this approach is often plagued by practice or translational barriers because of the mismatch between the scientific intervention and the demands of frontline practice [9]. Hybrid designs are increasingly being used to move science beyond an excessive attention to internal validity. Clinical-intervention hybrid designs are commonly employed on the efficacy/effectiveness continuum [9,30] and generally provide more relaxed internal controls to improve generalizability. Hybrid effectiveness/implementation (E-I) designs [31]are relatively new, with distinct methodological opportunities and challenges. Hybrid E-I designs address clinical effectiveness but target the methods and procedures necessary to deliver and sustain interventions in real-world care settings. As a prerequisite, hybrid E-I designs require a minimum of “indirect evidence” supporting a clinical intervention [31]. Without preexisting evidence to support the clinical intervention, implementation evaluation is premature.

**Figure 1:**
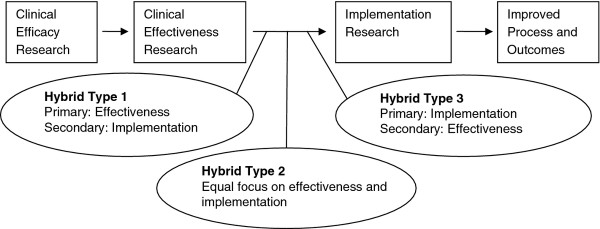
Hybrid effectiveness–implementation designs as part of the clinical research continuum.

As proposed by Curran *et al.*[31], hybrid E-I studies include a continuum of designs that move from effectiveness research with minimal implementation strategies (type 1) to designs where effectiveness and implementation are equally balanced (type 2) to a largely implementation approach with minimal focus on effectiveness outcomes (type 3) (Figure 1). The determination of which hybrid E-I design to choose is based on current literature and practice patterns.

Prototypical clinical and implementation research designs do not share many design features. Thus, a series of compromises are needed when creating and conducting a hybrid E-I study to balance the demands associated with scientific control or internal validity and factors associated with implementation, such as evaluation and improvement of processes and systems of care. Although compromise between these competing constructs may prove challenging, hybrid designs have the potential to (1) shorten the existing lag time between research discovery and uptake in care settings, (2) provide additional knowledge of clinical processes and system factors critical to adoption and long-term utilization, and (3) reduce cost by streamlining and combining elements of the traditional step-wise progression of research (efficacy to effectiveness to implementation) [31]. Most importantly, hybrid E-I designs that use strong and broad-reaching partnerships improve the collective shared vision among stakeholders and, ultimately, improve the odds that interventions developed will be adopted into practice.

Evaluation of hybrid E-I designs involves careful attention to the assessment of outcomes related to both clinical effectiveness and implementation success. RE-AIM is a commonly used framework to aid in addressing the unique challenges of blended E-I designs [32-36]. RE-AIM seeks to enhance evaluation and reduce the disparities between research, applied clinical practice, and sustainability of evidence-based practices over time [28,34] by focusing on five components: **R**each (the participation rate of the targeted population), **E**fficacy/**E**ffectiveness (the impact of the intervention on outcome criteria), **A**doption (number or proportion of sites/clinicians who provide the clinical intervention), **I**mplementation (intervention integrity, quality, and consistency of delivery), and **M**aintenance (sustainability of the intervention over time).

This protocol article describes a hybrid type 2 study, entitled “Cognitive Behavioral Therapy in Primary Care: Treating the Medically Ill.” Consistent with the hybrid E-I design [31] and informed by the RE-AIM framework [34], the project focuses on the effectiveness of bCBT for patient outcomes related to depression, anxiety, and physical health, while also seeking to simultaneously pilot test the acceptability, feasibility, and preliminary outcomes of a multifaceted implementation strategy to enhance patient engagement, as well as clinician adoption and fidelity. The article highlights study procedures and critical methodological decisions related to conducting a hybrid E-I study for bCBT.

## Methods

The Adjusting to Chronic Conditions Using Education, Support, and Skills (ACCESS) study was developed to examine the clinical effectiveness and implementation potential of an evidence-based, patient-centered bCBT intervention for depressed and/or anxious medically ill veterans. The project is being conducted within the PC-MHI programs of two large VA medical centers in the south central United States. The current study was approved by the Baylor College of Medicine Institutional Review Board (H-27082) and local VA Research and Development review committee. As such the study is in compliance with the Helsinki Declaration and all study participants provided informed consent including permissions for publication of this report.

ACCESS uses a patient-level, randomized design and focuses on veterans with chronic obstructive pulmonary disease (COPD) and/or heart failure (HF). COPD and HF are highly prevalent and burdensome to patients and the healthcare system [37-39]. These conditions are exacerbated by clinically elevated symptoms of depression and anxiety, which are common in approximately 50% of this patient population [40-43].

**Figure 2:**
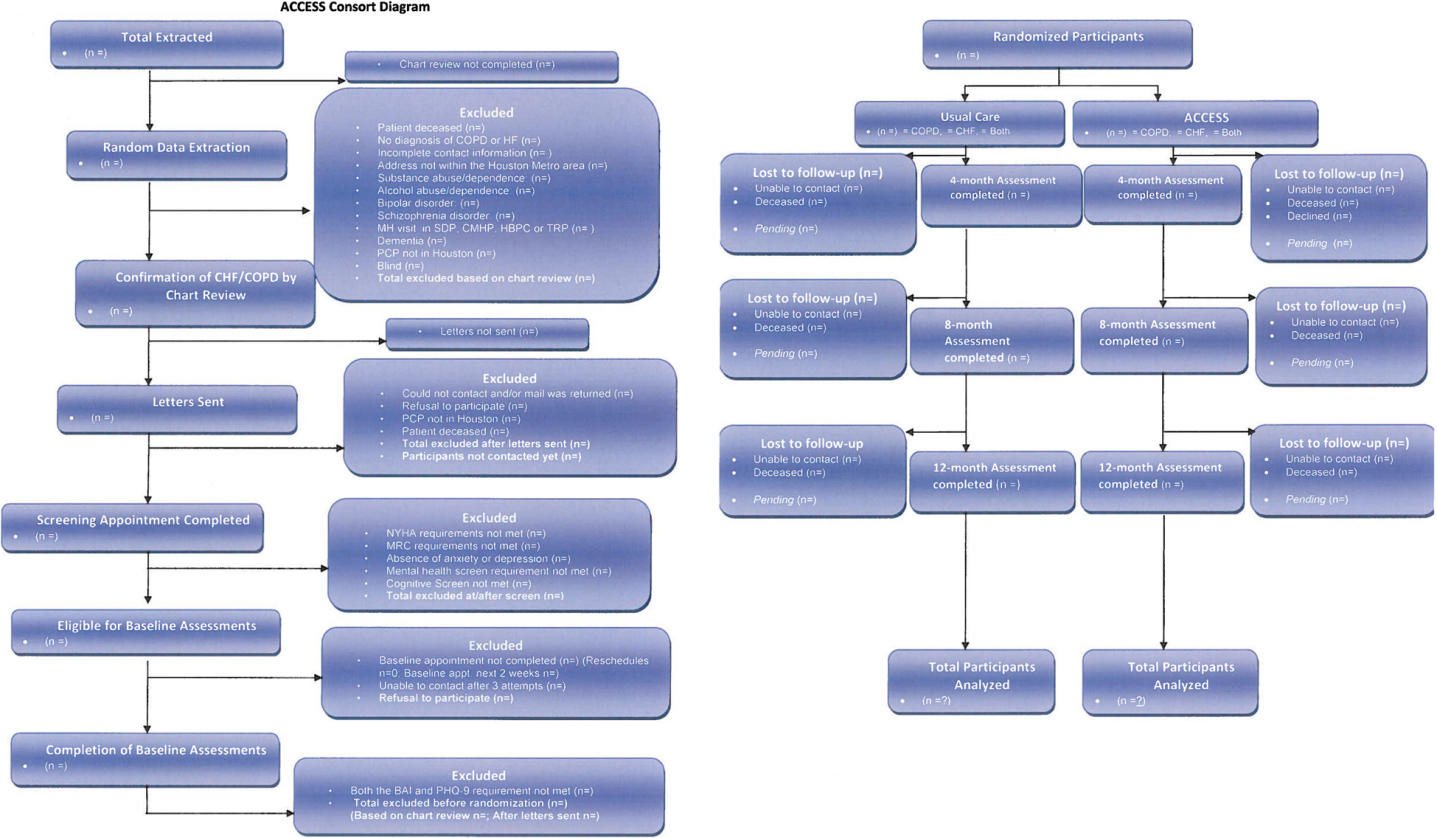
**Study CONSORT diagram illustrating flow of participants from recruitment to random assignment to follow-up.** ACCESS = Adjusting to Chronic Conditions Using Education, Support, and Skills; COPD = chronic obstructive pulmonary disease; HF = heart failure; MH = Mental Health; SDP = Substance Dependence Program; CMHP = Comprehensive Mental Health Program; HBPC = Home Based Primary Care; TRP = Trauma Recovery Program; PCP = primary care physician; NYHA = New York Heart Association; MRC = Medical Research Council Breathlessness Scale; BAI = Beck Anxiety Inventory; PHQ-9 = Patient Health Questionnaire-9.

ACCESS seeks to recruit 320 primary care patients with COPD and/or HF and comorbid clinically elevated symptoms of depression and/or anxiety, identified through VA databases and solicited using opt-out letters (see Consort Diagram in Figure 2). Of these, 120 patients will be randomized to usual care (UC) and provided with feedback about their depression and/or anxiety and encouraged to obtain services through their primary care provider. The remaining 200 patients will be randomized by blinded study staff to a bCBT intervention (*e.g.*, ACCESS intervention) provided by frontline mental health clinicians in two VA PC-MHI clinics. PC-MHI clinicians, who are mental health practitioners from various disciplines, including psychology, social work, nursing, and physician assistant backgrounds, will receive a comprehensive set of implementation interventions to enhance use and quality of the ACCESS intervention within their ongoing clinical care clinics. The collective implementation strategy to be pilot tested was created from the research team’s past experience in training clinicians in bCBT procedures and from the available implementation literature. The implementation strategy includes the following interventional components: (1) online clinician training; (2) audit and feedback of clinician session content, as provided by expert review; and (3) internal and external facilitation to support CBT use, including identification of system/clinic barriers.

The study has two primary purposes: (1) to determine the clinical effectiveness of the ACCESS intervention (bCBT) relative to UC on patient outcomes related to symptoms of depression and anxiety and disease-specific physical functioning and (2) to pilot test and examine the acceptability, feasibility, and preliminary outcomes of an implementation strategy designed to improve patient engagement and adherence, as well as intervention quality. Both effectiveness and implementation outcomes (see Table 1 for study objectives) will be assessed, using mixed methods involving qualitative procedures (*e.g.*, focus interviews and surveys conducted with patients and providers) and quantitative analyses (*e.g.*, intent-to-treat analyses and formal session fidelity ratings).

**Table 1:** Study outcomes and objectives

**Outcome**	**Objective**
**Effectiveness**	#1: To determine whether a bCBT treatment group as provided by VA PC-MHI clinicians is superior to a usual-care control group at post treatment and 8- and 12-month follow-ups, as measured by:
	a) depression and anxiety scores (Patient Health Questionnaire-9 and Beck Anxiety Inventory)
	b) cardiopulmonary disease outcomes (Chronic Respiratory Questionnaire and Kansas City Cardiomyopathy Questionnaire).
**Implementation**	#2: To assess bCBT adoption and fidelity, as measured by:
	a) bCBT patient engagement (one or more sessions) and adherence (four or more sessions)
	b) PC-MHI clinician bCBT adherence and competency ratings as evaluated by expert audio session reviews.

*bCBT* brief cognitive behavioral therapy, *VA* Department of Veterans Affairs, *PC-MHI* Primary Care-Mental Health Integration.

### Objective #1: clinical effectiveness of brief cognitive behavioral therapy

Comparative effectiveness trials suggest a general equivalence between CBT and other therapeutic modalities [44], with CBT being particularly well-suited for implementation within primary care settings, given its skill-based approach and ability to function with a restricted time and session framework [45,46]. Recent evidence suggests the general efficacy of bCBT for patients in primary care and patients with comorbid health conditions [17,18,47,48]. However, limited data exist on the real-world effectiveness of bCBT within the primary care setting, especially when used for the chronically medically ill [49].

### Clinical intervention

The ACCESS intervention is a manualized bCBT protocol that provides a flexible, patient-centered approach to increase patient engagement and adherence, while addressing both the mental and physical health needs of veterans with COPD and HF. ACCESS consists of six weekly treatment sessions and two brief (10- to 15-minute) telephone “booster” sessions within a four-month time frame. Participants are asked to attend the first session in person and can participate in subsequent sessions by telephone or in person.

The ACCESS intervention was developed based on prior studies and input from a variety of stakeholders, including patients, providers, PC-MHI directors, and national CBT experts [50]. Particular emphasis has been placed on maximizing intervention potency and minimizing session intensity and duration to maximize patient engagement and adoption within PC-MHI settings. Patients initially receive core modules focused on increasing awareness and controlling physical and emotional symptoms and subsequently are able to select skills from a series of module choices best aligned with their most pressing needs. Therapists follow a structured intervention manual, yet retain the ability to streamline and tailor the bCBT intervention collaboratively with patients when selecting treatment modules and goals. A patient workbook is provided to structure session content, guide telephone interactions, and provide opportunities to guide patient practice of skills between sessions.

Following an iterative developmental process [19,50], the ACCESS intervention was modified to be consistent with the PC-MHI model of the VA of brief four to six sessions of psychotherapy. ACCESS provides clinicians with structure and details that seek to ensure high fidelity to the intervention, while retaining flexibility in administration so as to mirror traditional psychotherapy practices. Data from a previously conducted open trial suggest that ACCESS improves both physical and emotional health outcomes and results in high engagement and adherence [19]. Detailed descriptive information about the content and processes of the ACCESS intervention can be found elsewhere [50].

### Identification and recruitment of participants

Study participants are being recruited from the Houston and Oklahoma City VA medical centers, using VA administrative databases to identify COPD and HF patients who received care in the prior year. Houston and Oklahoma City VAs were chosen for their size and integration of primary care mental health within the Veterans Integrated Service Network 16. Opt-out letters are mailed to participants to ensure the widest catchment possible. Interested participants complete a telephone screening process. Participants are included in the study if they are confirmed to have patient-reported functional limitations associated with their COPD and/or HF and have clinically significant symptoms of anxiety and/or depression. Participants are excluded only for clinical factors (*e.g.*, ongoing psychotherapy, concurrent specialty mental healthcare) or patient factors (*e.g.*, cognitive, bipolar, psychotic, or substance use disorders) that would render a PC-MHI bCBT intervention or PC-MHI treatment setting inappropriate [51]. Patients receiving psychotropic medications are not excluded, although they have their medication use monitored during the course of the study.

Sample size was established to ensure adequate power to detect differences in the primary patient outcomes for depression, anxiety, and physical health. To ensure 80% power to detect differences at the 0.05 level for all study measures, adjusting for repeated measures, a sample size of 180 randomized patients was necessary. However, this sample-size calculation was inflated because of the potential for intraclass correlations related to the clustering of patients within bCBT providers. Using an established inflation factor [52] and controlling for attrition of 25%, the final sample-size calculation required 200 participants for the ACCESS arm and 120 for UC (320 total). Unequal randomization, an accepted practice in clinical trials [53], was used to reduce unnecessary recruitment without significant impact on power. Randomization was stratified by medical condition (COPD only, HF only, both COPD and HF) and by site, using random block sizes of 5 or 10. Because of the higher number of patients in Houston, 200 of the 320 total patients will be randomized at Houston and 120 at Oklahoma City. Random allocation lists were generated using SAS statistical software (SAS Institute, Inc., Cary, NC, USA). Randomization numbers are drawn from envelopes stuffed by non-project staff and opened only after a participant is deemed fully eligible and ready for group assignment.

### Clinical effectiveness: outcome measures

Clinical effectiveness will be evaluated posttreatment (at 4 months) and at 8- and 12-month follow-ups by trained independent evaluators not associated with other aspects of the study. Assessment measures were selected to evaluate both physical and emotional health constructs related to COPD/HF patient-centered outcomes (Chronic Respiratory Questionnaire [54], Kansas City Cardiomyopathy Questionnaire [55]), anxiety [56], and depression (Patient Health Questionnaire-9 (PHQ-9) [57]). These effectiveness measures have strong psychometric properties and possess a high degree of external validity for clinical use in the primary care setting. For example, within the VA, the PHQ-9 is the standard measure for depression screening and intervention outcome evaluation [58]. Other study variables include demographic variables, COPD/HF severity, presence/absence of coexistent psychiatric and medical conditions, psychotropic medication use, and health-services use, including ambulatory and inpatient care, primary care, and specialty care. Treatment intensity (“dose”) will be measured by total number of bCBT sessions.

In addition to the above quantitative measures, semistructured-interview data are being collected from patients and providers regarding the impact of the intervention on physical and emotional health and identification of salient aspects of the treatment. Interviews will be conducted with all enrolled clinicians and a purposeful sample of 5 to 10 patients identified during the later stages of the trial. Patients are selected based on ACCESS clinician recommendations. Clinicians also refer ACCESS patients who recently completed treatment and might be able to share additional information about the intervention (whether positive or negative and regardless of treatment engagement).

### Clinical effectiveness: analyses

All quantitative analyses will be done on an intention-to-treat basis, and participants will be analyzed in the group to which they were randomized. Absolute differences in outcome measures will be examined between baseline and 12-month follow-up, and the effect sizes of the bCBT group will be compared with those of participants in the UC group. We will also examine effect sizes for the bCBT group baseline versus posttreatment and eight-month follow-up. To compare changes between the two groups over time, we will use longitudinal, mixed-model analyses that allow nesting of patients by medical center site.

Qualitative data will be collected using audiotaped interactions of the semistructured interview, transcribed and coded for themes, in combination with interviewer (field) notes. Using content-analysis methods, we will code textual data from the patients’ and providers’ perspectives to uncover patterns in the experiences of the bCBT intervention [59-62]. Coding and analysis will be conducted using formal qualitative-research methods. It is anticipated that qualitative data will provide details about the meaning of the intervention and its context within primary care that are unattainable using survey methods.

### Objective #2: implementation strategy and evaluation of adoption and implementation

Objective #2 seeks to pilot test and evaluate the acceptability, feasibility and preliminary outcomes of a comprehensive implementation strategy designed to increase bCBT engagement, adherence, and quality. The development and administration of these procedures are viewed as important to understanding the barriers and facilitators associated with access, adoption, and fidelity of bCBT. Given the developmental nature of this work, a case-study design was used without a clinician control group.

### Partnership development and implementation framework

Implementation planning for this study began with the construction of multiple stakeholder partnerships within the VA PC-MHI program. Study investigators with knowledge and prior practice experience in PC-MHI actively worked with PC-MHI clinicians and directors at both intervention sites to examine interests and needs related to evidence-based bCBT practices. The project team worked with these frontline stakeholders to identify needs and practice barriers to help inform the current project.

The ACCESS implementation strategy was informed by the Promoting Action on Research Implementation in Health Services (PARiHS) framework [63,64]. According to PARiHS, successful implementation (SI) is a function (f) of Evidence (E), Context (C), and Facilitation (F). *Evidence* refers to sources of knowledge and the importance of this knowledge as perceived by stakeholders. *Context* encompasses the environment or setting in which the improvement program is implemented. *Facilitation* refers to a technique by which an individual or group makes adoption or implementation easier for others, achieved through support in terms of attitudes, habits, skills, and ways of thinking and working.

### Implementation strategy

Based on stakeholder feedback and project-team experiences, the implementation strategy for this trial was developed to include three separate but interrelated interventions—online clinician training, clinician audit and feedback, and internal and external facilitation. Each element was developed within the PARiHS model and augmented by the available implementation literature. The project team focused on competing demands for depression treatment in the primary care setting—namely, constructs related to patients, providers, and the larger primary care clinical setting [65]. To address these three areas in accordance with the PARiHS framework, the implementation strategy included (1) online clinician training to address confidence and competency, as well as potential patient barriers related to presenting concerns, attitudes about mental health treatment, and initial goal-setting strategies to increase treatment engagement [45,66]; (2) audit and feedback of clinician session content, as provided by expert review [26,67] (audit and feedback was viewed as a critical element to not only assess treatment fidelity but also to assist clinicians by providing timely feedback about performance both positive and negative); and (3) internal and external facilitation to support CBT use, including identification of system/clinic barriers [46]. Facilitation that represents a package of well-known change strategies employed by the facilitator to address individual or site-specific issues at the right time to promote change adoption [46] was seen as the primary implementation intervention to address barriers at the system level, including difficulties with patient scheduling and provider time allowances for therapy.

In addition to these elements, ACCESS engages the participation of an advisory council consisting of various stakeholders from PC-MHI programs, as well as regional and national mental health leaders within the VA. Given the pilot nature of this implementation strategy, particular emphasis has been placed on understanding stakeholder perspectives, using formative (pre-implementation) and process (during implementation) evaluations such that the implementation interventions could be modified as needed during the trial.

### ACCESS clinicians

The study has targeted the inclusion of 12 PC-MHI clinicians between the two sites. The project team is working collaboratively with local stakeholders and clinic directors to identify clinicians best suited to provide psychotherapy services. Clinician enrollment is not influenced by prior psychotherapy or CBT knowledge and skill. However, clinicians are expected to have an interest in using bCBT in their daily practice and are asked to voluntarily participate and formally consent to participate. Nurses, social workers, psychologists, counselors, nurse practitioners, and physician assistants will potentially be included in the trial. Pretrial data will be collected for all study providers to help identify unequal entry-level bCBT expertise in care providers. Follow-up fidelity data will be collected throughout the project to document provider skill and intervention adherence.

### Implementation intervention #1: ACCESS (bCBT) online clinician training

All study therapists participate in a comprehensive bCBT training program based on methods developed in a prior study [45]. Print-based intervention materials (*e.g.*, clinician manual and patient workbook) provide a foundational knowledge of the intervention [50]. Print-based materials are augmented by an online training course (www.vaprojectaccess.org) consisting of narrated audio slides and audio vignettes to elaborate on critical intervention elements (detailed in a separate article—Cully *et al.*[66]).

### Implementation intervention #2: audit and feedback

Following the introductory training, clinicians receive audit and feedback on their first ACCESS therapy patient. Clinicians audiotape each session and have those audiotapes reviewed by a bCBT expert for adherence and skillfulness, using a standard rating scale [19,26,68]. Feedback from these evaluations is provided to the clinician after session 2 (completion of the core modules) and again at the conclusion of treatment. After completion of the first patient, clinicians continue to audiotape sessions, with audit and feedback occurring for a random selection and feedback on no less than two sessions every four months. While clinicians are not required to maintain minimal levels of adherence or skillfulness to remain in the trial (thus allowing for the evaluation of the intervention under real-world circumstances), those with scores falling below an *a priori* minimum performance standard are provided with increased frequency of audit and feedback.

### Implementation intervention #3: internal and external facilitation

External facilitation is provided to both intervention sites by bCBT experts/trainers as pilot tested by Kauth *et al.*[46]. The exact nature and content of external facilitation varies between sites, depending on clinician needs and logistical constraints. In general, external facilitators engage clinicians in regular individual or group meetings and less formally through telephone and email communication. The purpose of external facilitation is to provide clinicians with opportunities to address questions or concerns about the intervention, disseminate key information related to implementation, and create a sense of colleageality and community among the clinicians [69]. Although external facilitators will address topics related to ACCESS intervention procedures and strategies, they focus on assisting clinicians to adopt and maintain fidelity of bCBT in their daily practice [46].

Internal facilitation efforts, although not a robust implementation strategy for this study, consist of engaging PC-MHI directors to facilitate the adoption of bCBT within each site’s PC-MHI clinic. Study external facilitators work with PC-MHI directors (internal facilitators) to share information related to the project and encourage the directors to provide resources to clinicians to facilitate training and use of bCBT. External facilitators and study personnel also engage internal facilitators at both sites to streamline clinical processes and increase adoption, as dictated by the unique needs at each site.

### Study advisory council

A team of researchers and “end users,” including VA clinical managers and regional and national VA mental health leaders, have agreed to participate as study advisors to monitor progress and provide feedback on adoption and implementation of the bCBT intervention. The advisory council will meet semiannually throughout the study. During these meetings, the advisory council will receive updates on study progress, and notable occurrences will be discussed. The primary purpose of these meetings is to identify and address challenges and effective practice patterns to improve adoption of bCBT in other primary care settings within the VA. The advisory council also serves as an advocate for change and as a conduit for dissemination of study findings.

### Implementation evaluation plan and analyses

Engagement and adherence will be measured for all participants randomized into bCBT. Patient engagement is defined as the percentage of randomized patients that attend at least one CBT session. Adherence is defined as the completion of four or more active-treatment sessions. We will compare the percentage of patients with engagement and adherence by bCBT clinician and site and subsequently examine overall rates to those found in similar brief therapy trials [47]. The study advisory committee will review these outcomes and make recommendations as to the “clinical significance” of these findings relative to VA policy and current initiatives in PC-MHI.

We will also use clinician survey data to examine implementation success according to principles contained in the PARiHS framework, as detailed by Stetler *et al.*, 2011 [64]. Survey data will elicit provider Likert-style responses to domains related to consistency of ACCESS procedures with available evidence-based mental health treatments (PARiHS evidence) and suitability of ACCESS for PC-MHI (PARiHS context), as well as detailed questions eliciting feedback and perceptions of the various implementation interventions (PARiHS facilitation). Although not the focus of the study, amount of time spent conducting the implementation interventions will be collected.

Qualitative methods (individual interviews) with bCBT stakeholders (patients, clinicians, and clinic directors and managers) will be used to elicit information on perceptions of bCBT importance, potential for adoption, and potential barriers to implementation. Individual interviews will be conducted using a semistructured interview format and conducted by an experienced psychotherapist. The semistructured interview format will allow for flexibility to explore each individual stakeholder’s perspectives. These data, which are not attainable through traditional survey methods, will provide another level of data on acceptability, feasibility, and impact of the implementation interventions to help determine the potential for wider use of the implementation strategy in future trials.

Finally, taking advantage of the longer-term posttreatment patient follow-up period, we will examine clinician bCBT utilization rates six months postcompletion of all study implementation efforts. Using a self-report survey, we will measure frequency of bCBT procedures and techniques used by clinicians to document perceived practice changes during and after implementation.

## Discussion

Hybrid E-I designs have the potential to rapidly advance the utilization of evidence-based practices for complex mental health treatments such as bCBT. However, impact of these designs is strongly tied to the project team’s ability to form meaningful partnerships with stakeholders, including patients, clinicians, administrators, and policy makers. Effective partnerships are more likely to translate into a collaborative team-based approach and a shared vision. From this shared vision, the team can more effectively address the logistical and methodological challenges associated with administering interventions and changing practice patterns. Although the “upfront cost” of building these partnerships is significant, these collaborations are more likely to generate high-quality treatments that are feasible for frontline practice settings and may avoid common pitfalls associated with *post hoc* adaptations to efficacy-based interventions, especially the threat to validity that commonly occurs when translating evidence to practice [70]. Although hybrid designs may aid in mitigating the current science-to-service gaps [28], the application of these designs requires thoughtful planning and potentially difficult methodological compromises.

The ACCESS project began with grant preparations that engaged local, regional, and national stakeholders. Stakeholders unanimously agreed that bCBT interventions were needed for the PC-MHI setting and that clinicians would benefit from having additional resources to learn and apply these treatments. Given this shared vision, the project team worked actively with stakeholders to create a clinical intervention and implementation strategy that was viewed as feasible and meaningful. The project team was then able to focus on addressing the methodological challenges of blending a study with both effectiveness and implementation goals. For the ACCESS project, the team thought it critical to test a set of clinical and implementation interventions that would provide data applicable for the scientific, clinical, administrative, and policy communities. At its core, the ACCESS project seeks to provide knowledge that will lead to improvements in the provision of mental healthcare for the primary care setting.

The following sections provide examples of the important methodological compromises identified and addressed in the current study, focusing on the balance between internal validity and improvement of care processes. Consistent with other hybrid studies, ACCESS requires a longer-term study period (four years) and considerable financial and human capital resources. The trial also targets a large number of study participants (N = 320), which allows greater statistical power to examine mediators and moderators to balance the need for effectiveness and implementation outcomes simultaneously.

### Methodological challenge #1: patient identification, recruitment, and inclusion criteria

Traditional efficacy designs are conducted with strict internal controls and rigorous patient inclusion and exclusion criteria, often at the expense of restricting applicability to clinical practice. The current trial uses relaxed inclusion and exclusion criteria to more closely align study participants with typical patients seen by PC-MHI clinicians in their daily practice. The study also uses symptom (rather than diagnostic) assessment tools commonly used in PC-MHI.

Patient evaluations are conducted by independent evaluators and allow separation of the clinical and evaluation components of the trial. Most importantly, these evaluations serve as a method for obtaining detailed clinical information about patients, which can be used during secondary mediator and moderator analyses without posing unnecessary restrictions on participant inclusion criteria.

### Methodological challenge #2: clinical intervention and interventionists

Clinical interventions must possess replicable procedures but also have high levels of external validity and be feasible within the predetermined practice environment. The clinical intervention for the current trial was developed as a brief technique to closely align with PC-MHI care models. To increase flexibility and acceptability, the clinical intervention includes options for telephone sessions and allows clinicians and patients to select modules (content) to best address the patient’s individual needs. Although intervention flexibility is viewed as congruent with real-world practice, it also creates variation in the content and delivery. To retain these core treatment aspects while retaining moderate internal controls, the project uses comprehensive data-collection strategies, such as medical-record reviews and fidelity ratings of session content, to describe and statistically account for variability in treatment delivery in secondary analyses.

### Methodological challenge #3: implementation strategy and modification of clinic procedures

The immediate aim of the implementation strategy is to provide PC-MHI clinicians and clinics with the necessary support to conduct bCBT. In the current trial, a developmental implementation strategy is being pilot tested for acceptability, feasibility, and preliminary outcomes. Unlike the clinical intervention, which will remain unchanged throughout the study, the implementation interventions are conceptualized as developmental, given the potential for variability in practice patterns and needs within the two PC-MHI sites. In developing the implementation interventions, the project team (including stakeholders) targeted learning opportunities that could be feasibly embedded within the existing PC-MHI structure. We also sought to create objective procedures to be tested and refined for future use in a more comprehensive implementation trial. Lastly, the evaluation team intends to rely heavily on stakeholders and the advisory council to direct implementation efforts. As such, stakeholders will receive regular implementation updates, and we will elicit feedback from these groups to identify needed changes. The long-term implementation aim is to better understand the impact of a refined implementation strategy using a cluster randomized design. Ultimately, this work seeks to aid in the identification of best-practice guidelines for psychotherapy use in the primary care setting.

### Opportunities for knowledge transfer and dissemination of project results

Transfer of information, including research findings, training, and clinical-care practices from this study, will be ongoing and multifaceted. The dual approach of the hybrid type 2 design will allow the project to meaningfully target frontline practitioners and managers, national scientific research journals and professional organizations, as well as national, regional, and local VA and other academic hospital leaders and policy makers.

The project team intends to disseminate published training and intervention materials to frontline practitioners both within and outside the VA through the established ACCESS website (www.vaprojectaccess.org), as well as through focused dissemination channels within the VA, including regional and national distributions through the VA Veteran Integrated Service Networks and Central Office administration and leadership. The advisory council will assist in dissemination of study findings, both within and beyond the VA.

Preliminary data from the trial will focus on implementation and training process characteristics, the continued advancement of partnerships, and baseline clinical information on patient functioning. Final study data will address the study objectives related to clinical effectiveness of bCBT and the utility and success of the piloted implementation strategy to enhance bCBT adoption. Dissemination of these project findings will not only target traditional scientific publication avenues and national presentations but will also seek to inform clinical leaders at the national, regional, and local levels of the VA.

Although hybrid E-I designs are often complicated by methodological trade-offs, the opportunities for scientific and clinical practice advancements are significant. In the current trial, the dual focus on bCBT effectiveness and developmental implementation strategies will afford the project team a unique opportunity to simultaneously understand patient outcomes and clinical practices challenges. Further, given the structure of the research design, the project is likely to provide meaningful implementation data and scientific findings, regardless of the patient clinical outcomes. The implementation approach, constructed with active involvement of multiple stakeholders and end users, was broadly constructed around increasing the utilization of evidence-based psychotherapies within primary care. As such, the formative aspect of the implementation strategy will generate data that are distinct from the patient clinical-effectiveness aspects of the study and may inform future, more rigorous implementation studies.

## Summary

Corresponding to its desire to expand access to evidence-based mental health treatments for veterans, the VA is ideally situated and currently needs identification of feasible evidence-based treatments for use within the primary care setting. Hybrid designs like the one described in this study have the potential to rapidly advance the knowledge of both the clinical effectiveness of bCBT and the possible implementation of best practices necessary to support the use of this complex intervention in real-world care.

## Competing interests

The authors declare that they have no competing interests.

## Authors’ contributions

JAC is the principal investigator and contributed to study conception and design and manuscript preparation. MEAA, JM, and MRN helped with the design and acquisition of data related to clinician training and facilitation and contributed to manuscript preparation. ADN is a co-investigator, acted as a medical consultant, and contributed to study conception and design and manuscript preparation. MAS is a co-investigator and contributed to study conception and design, fidelity measurement, and manuscript preparation. KHS is a co-investigator and Oklahoma City site principal investigator and contributed to study conception and design, clinician training and facilitation in Oklahoma City, and manuscript preparation. MEK is a co-investigator and contributed to study conception and design and manuscript preparation. NJP is a co-investigator and contributed to statistical analyses, study conception and design, and manuscript preparation. MRK is a co-investigator and contributed to study conception and design and manuscript preparation. All authors read and approved the final manuscript.
